# Oligomeric β-Amyloid Suppresses Hippocampal γ-Oscillations through Activation of the mTOR/S6K1 Pathway

**DOI:** 10.14336/AD.2023.0123

**Published:** 2023-08-01

**Authors:** Ya-Li Wang, Jian-Gang Wang, Shuling Guo, Fang-Li Guo, En-Jie Liu, Xin Yang, Bingyan Feng, Jian-Zhi Wang, Martin Vreugdenhil, Cheng-Biao Lu

**Affiliations:** ^1^Department of Physiology and Pathophysiology, Henan International Joint Laboratory of Non-Invasive Neuromodulation, Xinxiang Medical University, Xinxiang, China.; ^2^Department of Cardiovascular Medicine, Luminghu District, Xuchang Central Hospital, Xuchang, China.; ^3^Department of Neurology, Anyang District Hospital of Puyang City, Anyang, China.; ^4^Department of Pathology, The First Affiliated Hospital of Zhengzhou University, Zhengzhou, China.; ^5^Key Laboratory of Translational Research for Brain Diseases, Shenzhen Institute of Advanced Technology, Chinese Academy of Sciences, Shenzhen, China.; ^6^Department of Pathophysiology, School of Basic Medicine and the Collaborative Innovation Center for Brain Science, Key Laboratory of Ministry of Education of China for Neurological Disorders, Tongji Medical College, Huazhong University of Science and Technology, Wuhan, China.; ^7^Department of Life Sciences, Birmingham City University, Birmingham, UK.; ^8^Department of Psychology, Xinxiang Medical University, Xinxiang, China.

**Keywords:** β-amyloid;, mTOR, S6K1, IPSC, gamma oscillation, APP/PS1

## Abstract

Neuronal synchronization at gamma frequency (30-100 Hz: γ) is impaired in early-stage Alzheimer's disease (AD) patients and AD models. Oligomeric Aβ_1-42_ caused a concentration-dependent reduction of γ-oscillation strength and regularity while increasing its frequency. The mTOR1 inhibitor rapamycin prevented the Aβ_1-42_-induced suppression of γ-oscillations, whereas the mTOR activator leucine mimicked the Aβ_1-42_-induced suppression. Activation of the downstream kinase S6K1, but not inhibition of eIF4E, was required for the Aβ_1-42_-induced suppression. The involvement of the mTOR/S6K1 signaling in the Aβ_1-42_-induced suppression was confirmed in Aβ-overexpressing APP/PS1 mice, where inhibiting mTOR or S6K1 restored degraded γ-oscillations. To assess the network changes that may underlie the mTOR/S6K1 mediated γ-oscillation impairment in AD, we tested the effect of Aβ_1-42_ on IPSCs and EPSCs recorded in pyramidal neurons. Aβ_1-42_ reduced EPSC amplitude and frequency and IPSC frequency, which could be prevented by inhibiting mTOR or S6K1. These experiments indicate that in early AD, oligomer Aβ_1-42_ impairs γ-oscillations by reducing inhibitory interneuron activity by activating the mTOR/S6K1 signaling pathway, which may contribute to early cognitive decline and provides new therapeutic targets.

## INTRODUCTION

Alzheimer's disease (AD) is a major threat to the well-being of the aging population. Extracellular β-amyloid (Aβ) precipitation and intracellular tau accumulation are hallmark pathologies, and hippocampus-associated spatial memory impairment is one of the early clinical symptoms in AD patients [1, 2]. Increased accumulation of aberrant Aβ has been attributed to suppressed autophagy [3]. However, in mild cognitive impairment (MCI) and early stages of AD, there is a pathological heterogeneity, and a significant proportion of non-demented aged subjects show typical AD pathologies [4, 5]. Therefore, the quest for the immediate cause of early cognitive decline in AD is imperative.

Synchronization of neuronal activity at frequencies in the gamma band (30-100 Hz: γ) provides a millisecond-precision timing matrix that facilitates inter-neuronal communication and determines the dynamic coupling of activity between brain areas [6, 7]. Hippocampal γ-oscillations of the local field potential measure the synchronization of neuronal activity, which correlates with indexes of working memory [8-10] and spatial memory [11]. γ-oscillations in the hippocampus have been associated with navigation [12] and hippocampus-based memory encoding/retrieval [13, 14].

Decreased γ-oscillations have been reported in AD animal models' prefrontal, lateral entorhinal, and olfactory cortex areas [9, 15, 16] and in AD patients [17]. A reduction in the global synchronization index in the γ band, a measure used to quantify synchronization between brain areas, correlates with cognitive decline in AD [18] and is already observed in MCI [19]. In transgenic AD models that produce high levels of Aβ, γ-oscillations are reduced at very early ages [16, 20-23]. Oligomeric Aβ causes a significant reduction in spontaneous network activity in the hippocampal area CA1 [24]. Fibrillar Aβ acutely degrades mouse hippocampal γ-oscillations in a concentration- and time-dependent manner [25, 26]. Interestingly, AD has been associated with neuronal hyper-excitability and epileptiform activity, primarily during reduced γ-oscillatory activity, which has been implicated in the progression of AD [27].

These studies suggest that impairments of synchronization at γ frequencies contribute to cognitive deficits in the early stages of AD [28, 29], and treatments that restore γ-oscillations could be a promising therapeutic target [23], but the molecular mechanisms underlying the Aβ-induced change of hippocampal γ-generating circuits remain elusive.

Gamma oscillations emerge from rhythmic inhibitory postsynaptic currents (IPSCs) that synchronize neuronal firing [30]. Because γ-oscillations are very energy-demanding [31], Aβ may reduce hippocampal γ-oscillations as the result of impairment of key metabolic processes involved in energy homeostasis. One option we pursued here is that Aβ affects energy homeostasis through changes in the mechanistic target of rapamycin (mTOR), a conserved Ser/Thr kinase that forms two multi-protein complexes known as mTOR complex 1 (mTOR1) and 2 (mTOR2)[32, 33], which weakens synaptic transmission [34] and mitochondrial function [35]. Evidence from AD in both patients and mouse models shows that hyper-activation of the mTOR pathway occurs at the early stages of AD [36, 37], and an increase in two mTOR downstream targets, p70 S6 kinase polypeptide 1 (S6K1) and eukaryotic translation initiation factor 4E (eIF4E)-binding proteins (4E-BPs), occurs already in MCI [38]. Reducing S6K1 expression improves spatial memory and synaptic plasticity in a mouse model of AD [39].

In the present study, we demonstrate that oligomeric Aβ causes impairment of hippocampal γ-oscillations through activating mTOR/S6K1 signaling.

## MATERIALS AND METHODS

### Animals

All animal experiments followed the "Principles of laboratory animal care" (NIH publication No. 86-23, revised 1985), as well as guidelines and regulations of the Ethics Committee of Xinxiang Medical College. C57BL/6J mice of either sex (3-4-week-old, unless specifically mentioned) were obtained from Beijing HFK Bioscience Co. The APP/PS1, double transgenic mice, were obtained from cross-breeding single transgenic mice expressing human APPK670N/M671L with single transgenic mice expressing human PS1M146L [40]. The mouse colonies were kept on a 12-hour light/dark cycle in temperature- and humidity-controlled rooms. Food and water were available ad libitum.

DNA samples were isolated from the tail tip of APP/PS1 transgenic mice at 3 weeks old for genotyping, using PCR with human APP primers and human PS1 oligo primers. For APP, the forward primer was 5’- GAC TGA CCA CTC GAC CAG GTT CTG -3’and the reverse primer was 5’- CTT GTA AGT TGG ATT CTC ATA TCC G -3’. For PS1, the forward primer was 5’-GAC AAC CAC CTG AGC AAT AC-3’, and the reverse primer was 5’-CAT CTT GCT CCA CCA CCT GCC-3’. APP/PS1 double transgenic mice displayed two target bands, while wild-type mice displayed no bands. APP/PS1 mice and wild-type mice were kept under standard conditions for up to 4-6 months in age.

### Slice preparation

Hippocampal slices were cut as previously described [41]. Animals were anesthetized by intraperitoneal injection of chloral hydrate (400 mg/kg). Horizontal brain slices (350 μm) containing the ventral hippocampus were cut at 4-5 °C in a cutting solution saturated with carbogen (95% O_2_ and 5% CO_2_) using a Leica VT1000S vibratome (Leica Microsystems UK, Milton Keynes, UK). The cutting solution contained (in mM): 225 sucrose, 3 KCl, 1.25 NaH_2_PO_4_, 24 NaHCO_3_, 6 MgSO_4_, 0.5 CaCl_2,_ and 10 glucoses (pH 7.4; 305 mOsm l^-1^). Immediately after slicing, sections were transferred and maintained in an interface chamber, continuously perfused with artificial cerebrospinal fluid (aCSF). The aCSF contained (in mM): 126 NaCl, 3 KCl, 1.25 NaH_2_PO_4_, 24 NaHCO_3_, 2 MgSO_4_, 2 CaCl_2_ and 10 glucoses (pH 7.4; 305 mOsm l^-1^), saturated with carbogen. The slices were allowed to equilibrate at room temperature for at least 30 minutes before being moved to the recording chamber, where they were placed at the interface between aCSF (4-5 ml/min) at 32 °C and warm moist carbogen that maintained a thin ﬁlm of aCSF covering the slice, to ensure applied substances could diffuse into the area recorded.

### Extracellular field potential recordings

Extracellular field potentials were recorded from the stratum pyramidal of area CA3, with aCSF-ﬁlled glass pipette recording electrodes (3-5 MΩ) [42]. Field potentials were ampliﬁed with Neurolog NL106 AC-coupled ampliﬁers (Digitimer, Welwyn Garden City, UK) and band-pass ﬁltered at 2-200 Hz with Neurolog NL125 ﬁlters (Digitimer). After the mains line noise was removed with Humbug noise eliminators (Digitimer), the signal was digitized and sampled at 2 kHz using a CED-1401 Plus (Cambridge Electronic Design, Cambridge, UK) and Spike-2 software (Cambridge Electronic Design).

Ten minutes after placing the slices in the recording chamber, kainate (100 nM) was added to the aCSF to induce γ-oscillations. Gamma oscillations were recorded from area CA3c, where oscillation amplitude is normally the largest and least affected by the faster intrinsic γ-oscillations in CA1[42].

Oscillation power was calculated from the power spectrum, generated by fast Fourier transforms over 60-s epochs (1Hz bin size, Hanning window, FFT size 2048). The summated power in the γ frequency range (set at 20-60 Hz for 32 °C): γ power, was used for quantiﬁcation of the γ-oscillation strength. The peak frequency was determined as the local maximum of the power spectrum in the γ frequency range. Waveform auto-correlograms were calculated over 60-s band-pass (10-200 Hz) ﬁltered epochs.

To ensure γ power was stable before drugs and/or Aβ peptide were added, the kainate-induced oscillatory activity was left to develop for at least 60 minutes. The average over the 5 minutes before the application, was taken as a baseline control. To quantify the effect of the drug or Aβ peptide on γ-oscillations, the average measure during the last 5 minutes of the application was normalized to the baseline control. In a separate set of experiments, the slices were pretreated with drugs for 10 minutes after 60 minutes in kainate, followed by 60 minutes of Aβ peptide addition. The average measure during the last 5 minutes of the Aβ peptide addition was normalized to the baseline control, to quantify the effect of the drug plus Aβ peptide on γ-oscillations. The power of γ-oscillations varies between slices and continues to grow gradually in kainate. To control for the effect of time, the changes induced by drugs or Aβ peptides were compared to the changes with time in a control group, where slices were treated with kainate only for 120 minutes, and the average measure over the last 5 minutes was normalized to the baseline measure.

### Patch-clamp recordings

Whole-cell patch-clamp recordings were conducted in a submerged recording chamber perfused with aCSF (3-4 ml/minute at 30 °C). Pyramidal CA3 neurons were visually identified with a 40x water-immersion objective in an upright microscope (FN1, Nikon, Tokyo, Japan) and recorded using the whole-cell patch voltage-clamp technique, using glass pipettes (3-5 MΩ) as previously described [43]. Membrane currents were recorded with a Multiclamp 700B patch-clamp amplifier (Molecular Devices, Sunnyvale, USA), filtered at 2 kHz with a low-pass Bessel filter, and then digitized and sampled at 10 kHz with a Digidata 1440 (Molecular Devices).

All neurons included in this study had a resting membrane potential below -55 mV. The series resistance was tested before and after completion of each recording by measuring the current transient elicited by a 10-mV hyperpolarizing voltage step, and recordings were not analyzed for neurons with access resistance > 25 MΩ or if series resistance deviated >20% from the initial value.

For the recording of spontaneous excitatory postsynaptic currents (sEPSCs), electrodes were filled with (in mM): 100 Cs-methanesulfonate, 10 CsCl, 10 HEPES, 0.2 EGTA, 4 Mg-ATP, 0.3 Na-GTP and 10 Na-phosphocreatine (pH was set to 7.4 with CsOH and osmolarity was set to 290-310 mOsm l^-1^ with sucrose), and the membrane potential was clamped at -70 mV. 10 µM bicuculline was added to the aCSF to abolish GABA_A_-mediated inhibitory synaptic currents.

For the recording of spontaneous inhibitory postsynaptic currents (sIPSCs), electrodes were filled with a CsCl-based internal solution containing (in mM): 120 CsCl, 30 Hepes, 0.2 EGTA, 2 MgCl_2_, 1.0 CaCl_2_, 4.0 Mg-ATP and 5 QX-314 bromide (pH was set to 7.2 with CsOH and osmolarity was set to 290-310 mOsm l^-1^ with sucrose). D(-)-2-amino-5-phosphonopentanoic acid (D-AP5; 50 μM) and 2, 3-Dioxo-6-nitro-1,2,3,4-tetrahydrobenzo[f]quinoxaline-7-sulfonamide disodium salt (NBQX; 10 μM) were added to the aCSF to block ionotropic glutamate receptor-mediated synaptic currents.

pClamp 10 software (Molecular Devices, Sunnyvale, CA, USA) was used to detect and measure synaptic events during 180 s epochs. EPSCs or IPSCs were detected on a running template (mean of ~20 events) with a well-defined baseline. Criteria for detecting spontaneous events were: exceeding a 3 pA threshold for longer than 2 ms, having a rise time shorter than 3 ms, and rising faster than the decay.

Aβ peptides or drugs were added to the incubation solution containing 100 nM kainate 30 minutes before the start of patch-clamp recordings. While incubating, slices were continuously supplied with aCSF saturated with carbogen.

### Drugs

Amyloid β protein fragment 1-42 (Aβ_1-42_; Sigma-Aldrich, Louis, USA), rapamycin (Tocris Cookson, Bristol, UK), [2-((4-(5-ethylpyrimidin-4-yl)piperazine-1-yl) methyl)-5-(trifluoromethyl)-1H-benzo[d]imidazole] (PF4708671, Tocris Cookson), 2-[(4-(3,4-dichlorophenyl)-thiazol-2-ylhydrazono)-3-(2-nitrophenyl)]propionic acid (4EGI-1, Tocris Cookson) and NBQX (Tocris Cookson) were dissolved in dimethyl sulphoxide (DMSO). Kainate (Sigma-Aldrich), bicuculline methiodide (Tocris Cookson), and D-APV (Tocris Cookson) were dissolved in water. All drugs were stored in the freezer at 1,000-10,000 times the working solution, diluted immediately before use, and applied to the aCSF.

### Aβ_1-42_ preparation

Aβ_1-42_ was prepared as described previously [44-46]. Initially, Aβ_1-42_ lyophilized powder was dissolved in 100% 1,1,1,3,3,3-Hexafluoro-2-Propanol (HFIP) at 1 mg/ml concentration and then incubated for 2 hours at room temperature. The Aβ_1-42_ solution was dried under a gentle stream of nitrogen gas and then dissolved in dimethyl sulphoxide (DMSO) under sonication for 10 minutes to obtain a stock concentration of 5 mM that was stored at -80 °C. For the Aβ_1-42_ monomer preparation, the Aβ_1-42_ stock solution was diluted in ACSF to the appropriate concentration for immediate application. For the Aβ_1-42_ oligomer preparation, 50 μM monomeric Aβ_1-42_ was incubated at 4 °C for up to 12 hours. After incubation, the Aβ_1-42_ solution was centrifuged at 15,000 g for 10 minutes at 4 °C, and the soluble Aβ_1-42_ in the supernatant was collected. For the fibrillar Aβ_1-42_ preparation, Aβ_1-42_ was diluted to 100 μM and incubated at 37 °C for 7 days. The Aβ_1-42_ solution was centrifuged at 5,000 g for 10 minutes at room temperature to remove the supernatant and collect the precipitation.

**Figure 1.**
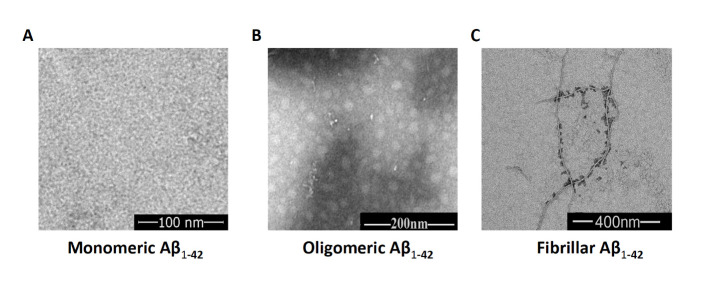
**The TEM images for monomers, oligomers, and fibrils of Aβ_1-42_**. (**A**) Example of a TEM image of Aβ_1-42_ monomer solution. (**B**) Example of a TEM image of soluble Aβ_1-42_ oligomers taken from the supernatant after incubation of Aβ_1-42_ monomer solution at 4 °C for 12 h. (**C**) Example of a TEM image of Aβ_1-42_ fibrils taken from the precipitate after incubating Aβ_1-42_ monomer solution at 37 °C for 7 days.

The biophysical and biological properties of Aβ_1-42_ were characterized by transmission electron microscopy (TEM) analysis [47-49]. Samples (5 μl) were diluted and deposited onto carbon-coated copper mesh grids for 5 minutes, and the liquid was absorbed with paper and negatively stained with 2% (w/v) uranyl acetate. Then, the sample grids were allowed to air dry. The samples were viewed with a JEOL JEM-1400 microscope (JEOL Ltd, Japan), and digital images were acquired with an Advanced Microscopy Techniques camera. Aβ_1-42_ oligomers appeared as fibril-free small globular structures that were <10 nm in diameter, and fibrillar Aβ_1-42_ showed long threads measuring >1 µm in length with some aggregated Aβs (Fig. 1). The morphological characteristics of these fibrils were identical to those of Aβ fibrillar structures, as has been reported [49, 50].

**Figure 2.**
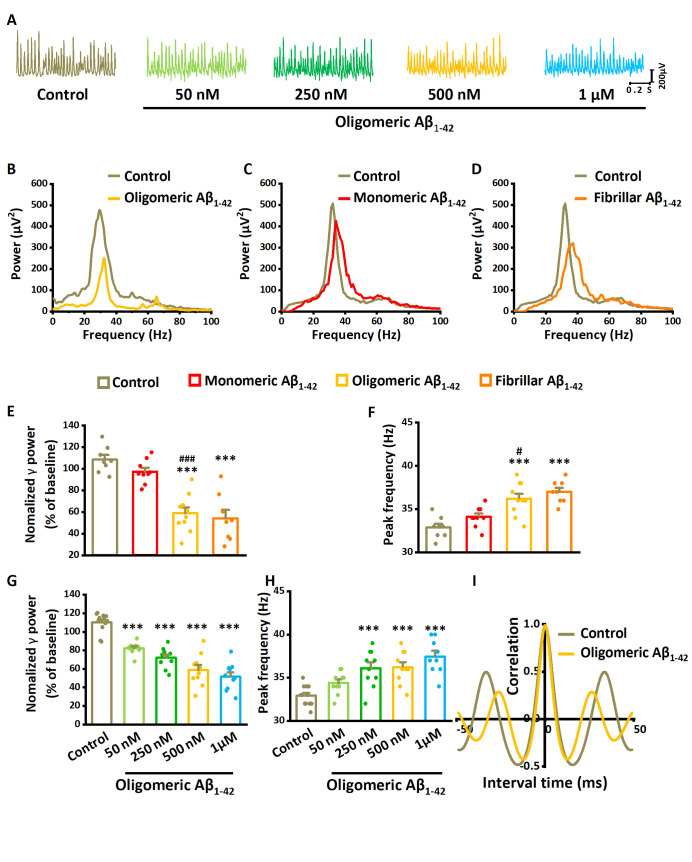
**The effect of Aβ_1-42_ on γ-oscillations**. (**A**) Example of oscillatory activity recorded in stratum pyramidale of CA3 before (brown trace) and 60 minutes after perfusion with 500 nM oligomeric Aβ_1-42_ (yellow trace). (**B**) Power spectra were taken from the recordings in (A), five minutes before application (baseline control: brown line) and 55-60 minutes after oligomeric Aβ_1-42_ application (yellow line). (**C**) Example of the power spectrum before (brown) and after 500 nM monomeric Aβ_1-42_ application (red line). (**D**) Example of the power spectrum before (brown) and after 500 nM fibrillar Aβ_1-42_ application (orange line). (**E**) γ-oscillation power as % of baseline control (100 nM kainate for 120 minutes), 500 nM monomeric Aβ_1-42_, 500 nM oligomeric Aβ_1-42_ and 500 nM fibrillar Aβ_1-42_. n= 8 for time-only control, n= 9 for monomeric Aβ_1-42_, n= 10 for oligomeric Aβ_1-42,_ n= 8 for fibrillar Aβ_1-42._ Significant differences with time-only control are indicated with **, *P*< 0.01, ***, *P*< 0.001. Differences between preparations are indicated with ^###^, *P*< 0.001 analyzed by unpaired t-test. (**F**) γ-oscillation peak frequency for the same groups, Details as in (E). (**G**) γ-oscillation power as % of baseline control for increasing concentrations of oligomeric Aβ_1-42_ and control. n=12 for time-only control, n=10 for 50 nM oligomeric Aβ_1-42_, n=10 for 250 nM oligomeric Aβ_1-42_, n=10 for 500 nM oligomeric Aβ_1-42_, n= 9 for 1μM oligomeric Aβ_1-42_. Data are presented as mean ± SEM. ***, *P*< 0.001, compared with time-only control, one-way ANOVA with Tukey’s post hoc test. (**H**) γ-oscillation peak frequency for control and increasing concentrations of oligomeric Aβ_1-42_. Details as in (G). (**I**) Auto-correlogram of the recording in A shows a 500 nM oligomeric Aβ_1-42_-induced reduction in oscillation regularity.

### Western blotting

Western blotting was performed by the methods established in our laboratory [43]. Briefly, for the preparation of total cell extracts, the dissected hippocampal area CA3 tissue was homogenized in lysis RIPA buffer containing 1% sodium dodecyl sulfate buffer in Tris-EDTA (pH7.4), 1× protease inhibitor cocktail (P8340, Sigma-Aldrich), 5mM NaF, and 1× phosphatase inhibitor cocktail (P2580, Sigma-Aldrich). The homogenate was centrifuged at 5,000 g for 10 minutes, the supernatant was collected, and total protein concentrations were measured using Bradford Assays. Proteins were electrophoretically separated in 12% SDS-PAGE gels and were transferred to a polyvinylidene fluoride membrane. The proteins were analyzed by Western blotting using antibodies against mTOR (1:500; Cell Signaling, 2972), phospho-mTOR (Ser 2448) (1:500; Cell Signaling, 2971), β-actin (1: 1,000; Abcam, ab6272). Membranes were then incubated with a horseradish peroxidenzyme-conjugatedated secondary antibody (1:2,000; Abcam, ab288151) to reveal the location of the protein bands. Immunoreactive bands were visualized by chemiluminescence using enhanced ECL reagents (BeyoECL Plus, Beyotime). Subsequently, X-ray ﬁlms were exposed to the membranes and then quantitatively analyzed by Image J software, which measures the intensity of protein bands after background subtraction. Relative protein level was calculated by normalizing phosphorylated form levels and total protein levels to the β-actin levels, respectively.

### Statistics

Data are expressed as mean ± standard error of the mean or medians ± min-max for non-normally distributed data. The normality of the distribution of data was assessed by the Shapiro-Wilk test. *N* indicates the number of hippocampal slices tested or cells recorded. Statistical comparisons between experimental conditions were made using the unpaired Student’s t-test or one-way ANOVA with Tukey’s post hoc tests for normally distributed data or the Mann-Whitney U test for non-normally distributed data. Signiﬁcance was assumed when *P* < 0.05.

## RESULTS

### Oligomeric Aβ_1-42_ impairs γ-oscillations in hippocampal slices

We first tested the effect of Aβ_1-42_ oligomer on kainate-induced γ-oscillations in mouse hippocampal slices. Gamma oscillations were induced by kainate (100 nM) for 60 minutes, after which slices were perfused for 60 minutes with different concentrations (50 nM-1 μM) of Aβ_1-42_ oligomer. At baseline, γ power was 1925 ± 331 µV^2^, and the peak frequency was 33.0 ± 0.4 Hz (n= 8 slices from 8 mice). Measurements of γ power were taken at the last 5 minutes before and after 60 minutes of application of Aβ_1-42_ oligomer. Aβ_1-42_ Oligomer decreased γ power by 45% of baseline control (t_16_= 6.949, *P*< 0.001, n=10 slices from 10 mice) and increased the peak frequency by 10% (t_16_= 4.302, *P*= 0.001, example in Fig. 2A and B).

We next tested the effects of monomeric Aβ_1-42_ and fibrillar Aβ_1-42_ on kainate-induced γ-oscillations. γ power at baseline was 2325 ± 312 µV^2^, and the peak frequency was 32.8 ± 0.3 Hz (n= 8 slices from 8 mice). Compared with the baseline control, monomeric Aβ_1-42_ (500 nM) decreased γ power by 10% (t_15_= 2.021, *P*=0.061, t-test, n= 9 slices from 9 mice, Fig. 2C and E), which was significantly less than the effect of 500 nM Aβ_1-42_ oligomer (t_16_= 6.949, *P*< 0.001, n= 10 slices from 10 mice). Monomeric Aβ_1-42_ increased the peak frequency by only 4% (t_15_= 2.112, *P*= 0.052, t-test, Fig. 2C and F). Compared with the baseline control, fibrillar Aβ_1-42_ (500 nM) decreased γ power by 50% (t_14_= 6.134, *P*< 0.001, t-test, n= 8 slices from 8 mice, Fig. 2D and E), which was not different from the effect of 500 nM Aβ_1-42_ oligomer (t_16_= 0.508, *P*= 0.619). Fibrillar Aβ_1-42_ increased the peak frequency by 13% (t_14_= 6.454, *P*< 0.001, t-test, Fig. 2D and F). These results suggest that the oligomeric and fibrillar forms of Aβ_1-42_ are more potent in suppressing and accelerating γ-oscillations than monomeric Aβ_1-42_, as reported previously [25]. However, since it is unlikely that large fibrils can diffuse into the tissue through the extracellular space [51], the effect of the fibrillar Aβ_1-42_ is likely due to the formation of oligomers from fibrils [52].

To quantify the effect of different concentrations of oligomeric Aβ_1-42_ on γ-oscillations, comparisons were made with the effect of a time-only control: the change in γ power after application of 100 nM kainate for 120 minutes (110% of baseline). Compared with the time-only control (n= 12 slices from 12 mice), oligomeric Aβ_1-42_ decreased γ power by 25% at 50 nM (*P*< 0.001, n(Aβ)= 10 slices from 10 mice), 35% at 250 nM (*P*< 0.001, n= 10 slices from 10 mice), 47% at 500 nM (*P*< 0.001, n= 10 slices from 10 mice) and 53% at 1 μM (F_4,50_= 38.77, *P*< 0.001, one-way ANOVA with Tukey’s post hoc test, n= 9 slices from 9 mice, Fig. 2G). Oligomeric Aβ_1-42_ increased the γ-oscillation peak frequency by 5% (50 nM, *P*= 0.269), 10% (250 nM, *P*< 0.001), 10% (500 nM, *P*< 0.001) and 14% (1μM, F_4,50_= 11.34, *P*< 0.001, one-way ANOVA with Tukey’s post hoc test, Fig. 2H). γ oscillations were very regular at baseline, reflected by a peak of 0.48 ± 0.03 at 30 ± 1 ms in the autocorrelogram. After applying 500 nM oligomeric Aβ_1-42_, the autocorrelation peak (at 28 ± 1 ms) was reduced to 0.33 ± 0.03 (t_20_= 3.38, *P*= 0.003, n= 21 slices from 21 mice, Fig. 2I). The reduction in autocorrelation indicates that in addition to the reduced oscillation strength, the γ cycle length was less regular after oligomeric Aβ_1-42_ exposure.

**Figure 3.**
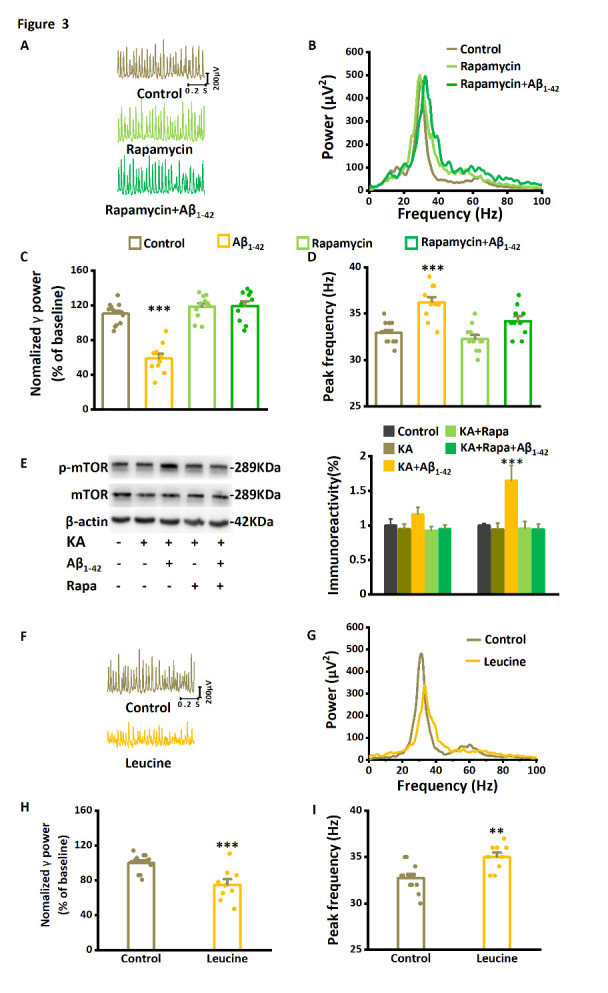
**The mTOR inhibitor rapamycin prevents Aβ_1-42_-induced effects on γ-oscillations and suppresses Aβ_1-42_-induced mTOR hyperactivation**. (**A**) Example traces of γ-oscillations for baseline control, after application of rapamycin-only, and after rapamycin plus Aβ_1-42_ (dark green trace). (**B**) Example of power spectra of γ-oscillations with the application of Aβ_1-42_ and rapamycin plus Aβ_1-42_. (**C**) γ-oscillation power as % of baseline control, in the presence of Aβ_1-42_, rapamycin only, or rapamycin with Aβ_1-42_. n= 12 for time-only control, n= 10 for Aβ_1-42_, n= 11for rapamycin only, n= 10 for rapamycin with Aβ_1-42_. (**D**) The effect of rapamycin and Aβ_1-42_ on the γ-oscillation peak frequency. Details as in (C). (**E**) Example of Western blot quantification of mTOR and the serine 2448 phosphorylated mTOR (p-mTOR). n= 9 mice for each group. (**F**) Example traces of γ-oscillations for baseline control and after application of leucine. (**G**) Example of power spectra of γ-oscillations for baseline control and after application of leucine. (**H**) γ-oscillation power as % of baseline control, in the presence of leucine. n= 12 for time-only control and n= 9 for leucine. The effect of leucine on the γ-oscillation peak frequency. Details as in (G). Data are presented as mean ± SEM. *, *P*< 0.05, **, *P*< 0.01, ***, *P*< 0.001, compared with time-only control, analyzed by one-way ANOVA with Tukey’s post hoc test in C, D, H and I. *, *P*< 0.05, compared with time-only control, analyzed by unpaired t test in E.

Since 500 nM oligomeric Aβ_1-42_ is in the range of pathological concentrations observed in brain tissue of AD patients [53], and was not significantly different from that of 1 µM (Turkey test *P*= 0.841), this concentration was used for the subsequent investigations of the cellular and network mechanisms underlying the acute oligomer Aβ_1-42_-induced impairment of γ-oscillations. The term Aβ_1-42_ is used for 500 nM oligomer Aβ_1-42_ in the remainder of this paper.

To study whether the Aβ_1-42_-induced reduction of γ-oscillations is specific to kainate-induced γ-oscillations, we tested the effect of Aβ_1-42_ on a different *in vitro* model of γ-oscillations. Using the same approach as for kainate-induced γ-oscillations, Aβ_1-42_ reduced γ power of activity induced by carbachol (10 µM) to 47% of control power (the control power was 105% of baseline, t_8_= 6.80, *P*< 0.001, data not shown). Aβ_1-42_ increased the peak frequency by 7% (t_8_= 3.80, *P*= 0.005, data not shown). These data suggest that oligomeric Aβ_1-42_ impairs γ-oscillations in hippocampal CA3 irrespective of the *in vitro* model.

**Figure 4.**
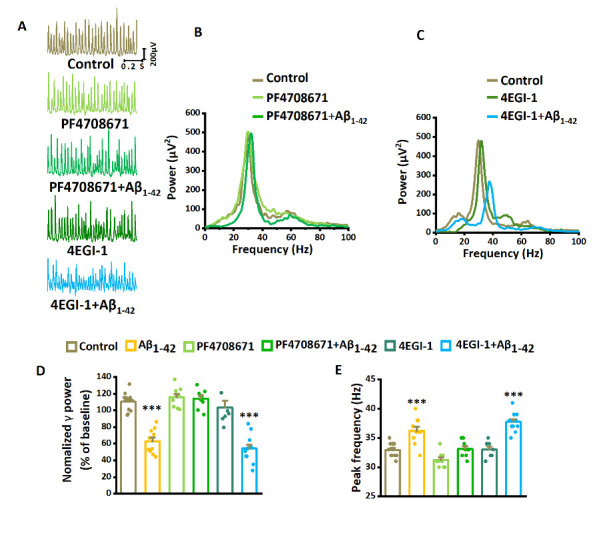
**The mTOR-mediated suppression of γ-oscillations requires S6K1 activation rather than eFI4E-eIF4G interactions**. (**A**) Example traces of γ-oscillations without and with Aβ_1-42_ exposure during PF4708671 application and 4EGI-1 application. (**B**) Example of power spectra of γ-oscillations without and with Aβ_1-42_ exposure during PF4708671 application. (**C**) Example of power spectra of γ-oscillations without and with Aβ_1-42_ exposure during 4EGI-1 application. (**D**) γ-oscillation power as % of baseline control, in the presence of Aβ_1-42_, PF4708671 only, PF4708671 with Aβ_1-42_, or 4EGI-1 only, 4EGI-1 with Aβ_1-42_. n= 12 for control, n= 10 for Aβ_1-42_, n= 9 for PF4708671 only, n= 9 for PF4708671 with Aβ_1-42_, n= 7 for 4EGI-1 only, n= 12 for 4EGI-1 with Aβ_1-42_. (**E**) Effect of PF4708671, 4EGI-1, and Aβ_1-42_ on γ-oscillation peak frequency. Details as in (D). Data are presented as mean ± SEM. ***, *P*< 0.001, compared with control, one-way ANOVA with Tukey’s post hoc test.

### Activation of mTOR/S6K1 signaling mediates Aβ_1-42_-induced impairment of γ-oscillations

Involvement of the mechanistic target of rapamycin (mTOR) activation in AD has been shown previously [36, 54]. To explore the potential role of mTOR in modulating γ-oscillations, we tested the effect of inhibition and activation of mTOR on γ-oscillations.

Slices were pretreated with mTORC1 inhibitor rapamycin (100 nM for 10 min) and then perfused with Aβ_1-42_ and rapamycin for 60 minutes. Compared with the reduction of γ power induced by Aβ_1-42_ only (by 47% of the control, n= 10 slices from 10 mice Aβ_1-42_ only), pretreatment with rapamycin prevented the effect of Aβ_1-42_ on γ power (increased by 12% compared with baseline control, *F*_3,42_= 37.01, *P*= 0.369, one-way ANOVA with Tukey’s post hoc test, n= 10 slices from 10 mice, Fig. 3A-C). Rapamycin also prevented the increase in peak frequency of γ-oscillations (increased by 4% compared with the baseline control, *F*_3,42_= 13.56, *P*= 0.217, Fig. 3D). To control the effects of rapamycin itself, we tested the effect of rapamycin only for 60 minutes. Rapamycin-only treatment had no significant impact on the γ power (increased by 4% compared with the control, *F*_3,42_= 37.01, *P*= 0.158, one-way ANOVA with Tukey’s post hoc test, Fig. 3A-C) or on the peak frequency (decreased by 3% compared with the baseline control, F_3,42_= 13.56, *P*= 0.742, Fig. 3D).

To examine whether mTOR was activated by Aβ_1-42_, CA3 tissue was dissected for Western blotting after electrophysiological recordings. As shown in Figure 3E, total mTOR levels were not different between control and Aβ_1-42_ treatment (F_4,14_= 1.086, *P*= 0.414, one-way ANOVA with Tukey’s post hoc test, each sample collected from 3 mini slices of hippocampal CA3 region of 3 mice, n= 3 independent experiments from 9 mice). However, Aβ_1-42_ treatment increased serine-2448 phosphorylation of mTOR by 65% (F_4,14_= 6.74, *P*= 0.021). Interestingly, the pretreatment with rapamycin prevented the effect of Aβ_1-42_ on serine-2448 phosphorylation of mTOR (F_4,14_= 6.74, *P*= 0.013). The data suggest that Aβ_1-42_ activates mTOR without affecting total mTOR expression and that rapamycin prevents Aβ_1-42_-induced activation.

**Figure 5.**
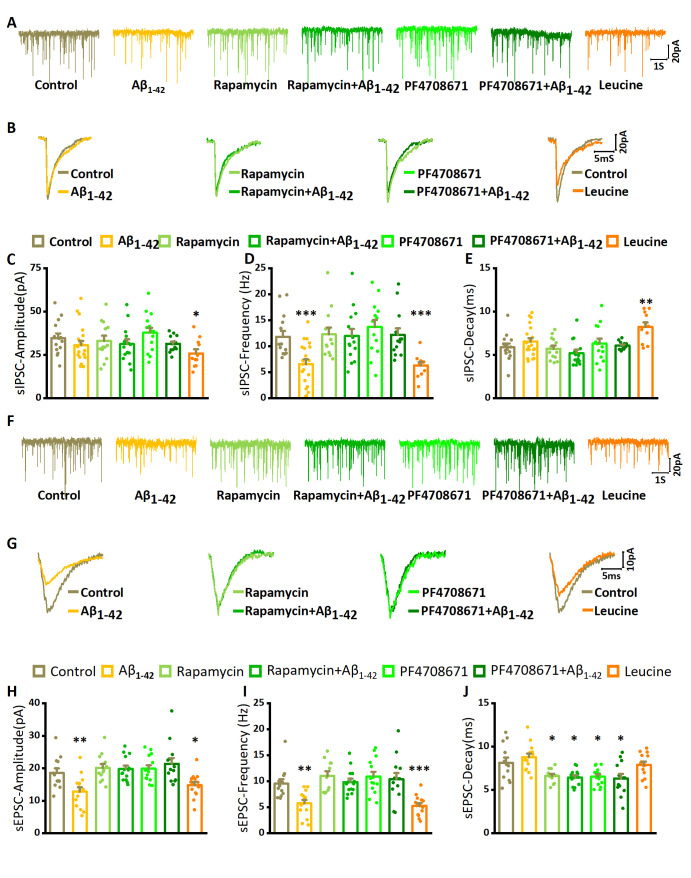
**The effect of Aβ_1-42_ on spontaneous IPSCs and EPSCs**. (**A**) Examples of IPSCs recorded in kainate aCSF only (control) after addition of Aβ_1-42_ exposure, in the presence of rapamycin or PF4708671, and after leucine application. (**B**) Waveform averages of the recordings in (A). (**C**) sIPSC amplitude of control (n= 14 from 5 mice), in the presence of Aβ_1-42_ (n= 20 from 7 mice), rapamycin only (n= 13 from 7 mice), rapamycin with Aβ_1-42_ (n= 14 from 5 mice), or PF4708671 only (n= 14 from 6 mice), PF4708671 with Aβ_1-42_ (n= 13 from 5 mice), or leucine only (n= 11 from 5 mice). Data are presented as mean ± SEM. *, *P*< 0.05, compared with control, unpaired Student’s t-tests. (**D**) sIPSC frequency for the same conditions and with same details as in (C). ***, *P*< 0.001, compared with control. (**E**) sIPSC decay time for the same conditions and with same details as in (C). **, *P*< 0.01, compared with control. (**F**) Examples of EPSCs recorded in kainate aCSF only (control) after addition of Aβ_1-42_ exposure, in the presence of rapamycin or PF4708671, and after leucine application. (**G**) Waveform averages of the recordings in (F). (**H**) sEPSC amplitude of control (n= 12 from 5 mice), in the presence of Aβ_1-42_ (n= 14 from 6 mice), rapamycin only (n= 14 from 5 mice), rapamycin with Aβ_1-42_ (n= 13 from 6 mice), or PF4708671 only (n= 12 from 5 mice), PF4708671 with Aβ_1-42_(n= 14 from 6 mice), or leucine only (n= 14 from 7 mice). Data are presented as mean ± SEM. *, *P*< 0.05; **, *P*< 0.01, compared with control, unpaired Student’s t-tests. (**I**) sEPSC frequency for the same conditions and with same details as in (H). ***, *P*< 0.001, compared with control. (**J**) sEPSC decay time for the same conditions and with same details as in (H).

**Table 1:** Effect of mTOR/S6K1 on spontaneous IPSC in hippocampal CA3.

Drug	N	Rm (GΩ)	Amplitude (pA)	*P*(t-test with control)	Frequency (Hz)	*P*(t-test with control)	Decay time (ms)	*P*(t-test with control)
**Control**	14(5)	193±17	34.7±2.7	–	11.8±1.1	–	5.9±0.4	–
**Aβ_1-42_**	20(7)	201±15	30.5±2.9	0.32	6.6±0.9	0.001***	6.5±0.6	0.408
**leucine**	11(5)	194±16	25.8±2.4	0.025*	6.3±0.7	<0.001***	8.2±0.7	0.007**
**rapamycin**	13(5)	198±15	33.0±3.0	0.678	12.4±1.4	0.756	5.7±0.4	0.747
**rapamycin + Aβ_1-42_**	14(5)	196±18	30.5±2.8	0.292	11.9±1.9	0.946	5.2±0.4	0.244
**PF4708671**	14(6)	208±19	37.9±3.6	0.49	13.7±1.2	0.4	6.3±0.6	0.552
**PF4708671 + Aβ_1-42_**	13(5)	207±16	31.4±1.3	0.288	12.2±1.7	0.85	6.1±0.1	0.684

Because mTOR inhibition prevented Aβ_1-42_-induced impairment of γ-oscillations, we predicted that increasing mTOR activity would mimic the effect of Aβ_1-42_ on γ-oscillations. Indeed, the mTOR activator leucine (3 μM, application for 60 min) decreased γ power and increased the peak frequency (Fig. 3F, G). Leucine decreased γ power by 25% (75.0±6.3% of the baseline control, compared with the baseline control, t_19_= 3.868, *P*= 0.001, n= 12 slices from 12 mice for control, n= 9 slices from 9 mice for leucine, Fig. 3H). It increased the peak frequency by 7% (35.0±0.5 Hz, compared with 32.8±0.4 Hz in the baseline control, t_19_= 3.506, *P*= 0.002, Fig. 3I). These data suggest that rapamycin-sensitive mTOR activity is both necessary and sufficient for oligomer Aβ_1-42_-induced impairment of γ-oscillations.

Ribosomal protein S6 kinase beta-1(S6K1), a protein kinase downstream of mTORC1, is activated in AD [55] and MCI [38]. Pretreatment of the slices with the S6K1 inhibitor PF4708671 (20 μM for 10 min), followed by perfusion with Aβ_1-42_ and PF4708671 for 60 minutes, did not affect γ power (increased by 5%, compared with the baseline control, F_3,39_= 34.39, *P*= 0.935, one-way ANOVA with Tukey’s post hoc test, n= 9 slices from 9 mice for PF4708671+ Aβ_1-42_, Fig. 4A, B, D), or the peak frequency of γ-oscillations (increased by 1%, compared with the baseline control, F_3,39_= 25.95, *P*= 0.992, Fig. 4E). To control the effects of PF4708671 itself, we tested the impact of PF4708671 only for 60 minutes. The PF4708671-only treatment did not affect γ power (increased by 3% compared with the control, F_3,39_= 34.39, *P*= 0.836, one-way ANOVA with Tukey’s post hoc test, n= 9 slices from 9 mice for PF4708671, Fig. 4A, B, D) or the peak frequency (decreased by 5% compared with the control, F_3,39_= 25.95, *P*= 0.105, Fig. 4E). These data suggest that Aβ_1-42_ suppresses γ-oscillations by activating the mTOR/S6K1 pathway.

Alternatively, mTORC1 activation could affect γ-oscillations through inhibition of eIF4E, which is elevated in AD [56] and MCI [38]. Activation of mTORC1 triggers the initiation of cap-dependent mRNA translation via phosphorylation of 4E-BPs [57]. The mTOR-dependent phosphorylation of 4E-BPs causes the release of eIF4E and its subsequent binding to eIF4G and permits the formation of the eIF4F initiation complex and subsequent cap-dependent protein synthesis [58]. To determine whether eIF4E-eIF4G interactions are required for the suppression of γ-oscillations by Aβ_1-42_, we tested the effect of the eIF4E-eIF4G interaction inhibitor 4EGI-1. 4EGI-1 (50 μM, application for 60 min) had no significant effect on γ power (decreased by 6%, compared with the baseline control, *F*_3, 40_= 29.70, *P*= 0.835, n= 12 slices from 12 mice for control, n= 7 slices from 7 mice for 4EGI-1, Fig. 4A, C, D) or the peak frequency (increased by 1%, compared with the baseline control, *F*_3, 40_= 22.31, *P*= 0.991, Fig. 4E). Pretreatment of the slices with 4EGI-1 (50 μM for 10 min), followed by perfusion with 4EGI-1 and Aβ_1-42_ for 60 minutes, decreased γ power (reduced by 51%, compared with the baseline control, F_3, 40_= 29.70, *P*< 0.001, n= 12 slices from 12 mice for 4EGI-1+ Aβ_1-42_, Fig. 4A, C, D), and increased the peak frequency of γ-oscillations (increased by 15%, compared with the baseline control, F_3, 40_= 22.31, *P*< 0.001, Fig. 4E). The effect of Aβ_1-42_ on γ power in the presence of 4EGI-1 was not different from that of Aβ_1-42_ alone (*P*= 0.929). The lack of impact of eIF4E inhibition confirms the importance of the mTOR/S6K1 pathway.

### Oligomeric Aβ_1-42_ affects EPSC and IPSC properties through the mTOR/S6K1 pathway

γ oscillations emerge from the rhythmic, mutual interactions of pyramidal neurons and interneurons in the local network [30], and extracellularly recorded γ-oscillations reflect mainly the amplitude and synchronicity of rhythmic IPSCs. To assess whether the Aβ_1-42_-induced impairment of γ-oscillations can result from the changes in synaptic properties and/or synchronicity, we tested the effect of Aβ_1-42_ on EPSCs and IPSCs, recorded in CA3 pyramidal neurons in a kainate-activated neuronal network (Fig. 5).

Hippocampal slices were treated with kainate (100 nM) or kainate plus Aβ_1-42_ for at least 30 minutes before pyramidal cells were recorded in whole-cell patch voltage-clamp mode. In submerged slices, kainate caused increased activity, but did not generate γ-oscillations. Spontaneous IPSCs were pharmacologically isolated and were inward at -70 mV (Fig. 5A). Aβ_1-42_ reduced the IPSC frequency by 45% (compared with a control frequency of 11.8±1.1 Hz, t_32_= 3.62, *P*= 0.001, n= 14 neurons from 5 mice for control group, n= 20 neurons from 7 mice for Aβ_1-42_ only, Fig. 5D, Table 1) but did not affect the IPSC amplitude (the control amplitude was 34.7±2.7 pA, Fig. 5C, Table 1) or the IPSC decay time (5.9±0.4 ms in control, Fig. 5E, Table 1). Aβ_1-42_ reduced the EPSC amplitude by 31% (compared with a controll amplitude of 18.5±1.4 pA, t_24_= 2.92, *P*= 0.007, n= 12 neurons from 5 mice for control group, n= 14 neurons from 6 mice for Aβ_1-42_ only, Fig. 5F and H, Table 2), and the EPSC frequency by 40% (the control frequency was 9.5±0.9 Hz, t_24_= 3.53, *P*= 0.002, Fig. 5F and I, Table 2), but did not affect the EPSC decay time (8.1±0.6 ms in control, Fig. 5G and J, Table 2).

**Table 2:** Effect of mTOR/S6K1 on spontaneous EPSC in hippocampal CA3.

Drug	N	Rm (GΩ)	Amplitude (pA)	*P*(t-test with control)	Frequency (Hz)	*P*(t-test with control)	Decay time (ms)	*P*(t-test with control)
**Control**	12(5)	187±10	18.5±1.4	–	9.5±0.9	–	8.2±0.6	–
**Aβ_1-42_**	14(6)	218±20	12.8±1.3	0.007**	5.7±0.7	0.002**	8.8±0.4	0.38
**leucine**	14(7)	193±10	14.8±0.9	0.037*	5.3±0.5	<0.001***	7.9±0.4	0.741
**rapamycin**	14(5)	215±24	19.9±1.1	0.422	10.9±1.0	0.257	6.5±0.3	0.04*
**rapamycin + Aβ_1-42_**	13(6)	201±17	21.4±1.7	0.471	10.4±1.2	0.777	6.3±0.5	0.013*
**PF4708671**	12(5)	215±17	20.1±1.2	0.457	11.0±0.9	0.321	6.6±0.3	0.021*
**PF4708671 + Aβ_1-42_**	14(6)	209±8	19.8±1.0	0.225	9.8±0.6	0.569	6.4±0.2	0.035*

Because the Aβ_1-42_-induced impairment of γ-oscillations was involved in mTOR activation, we predicted that rapamycin would prevent the Aβ_1-42_-induced changes in IPSC and EPSC properties. Hippocampal slices were pretreated with 100 nM rapamycin or rapamycin plus Aβ_1-42_ in the present with 100 nM kainate for at least 30 minutes. Rapamycin alone did not affect IPSC or EPSC properties (Fig. 5, Table 1, 2). After pretreatment with rapamycin for 10 min, adding Aβ_1-42_ had no effect on the amplitude or frequency of IPSCs and EPSCs (Fig. 5, Table 1, 2), but decreased the decay time of EPSCs by 20% (*t*_24_= 2.69, *P*= 0.013, Fig. 5, Table 1, 2).

Because leucine could mimic the effect of Aβ_1-42_ on γ-oscillations, we predicted that leucine would reduce both IPSC and EPSC frequency and EPSC amplitude. Using the same approach as with the application of Aβ_1-42_, leucine (3 μM) was applied with 100 nM kainate to slices for 30 minutes before the whole-cell recordings were started. Compared with the control, leucine decreased the IPSC frequency by 47% (*t*_23_= 3.96, *P*< 0.001, n=11 neurons from 5 mice, Fig. 5A and D, Table 1) and increased the IPSC decay time by 40% (*t*_23_= 2.99, *P*= 0.007, Fig. 5B and E, Table 1). The IPSCs amplitude was reduced by 26% (*t*_23_= 2.40, *P*= 0.025, Fig. 5A and C). Leucine decreased the EPSC frequency by 45% (*t*_24_= 4.36, *P*< 0.001, n=14 neurons from 7 mice, Fig. 5F and I, Table 2) and reduced the EPSC amplitude by 20% (*t*_24_= 2.20, *P*= 0.037, Fig. 5F and H). Leucine did not affect the EPSC decay time (Fig. 5G and J, Table 2).

Since PF4708671 could prevent the effect of Aβ_1-42_ on γ-oscillations, we tested whether S6K1 activation was involved in the effect of Aβ_1-42_ on IPSC and EPSC properties. As described for rapamycin, hippocampal slices were pretreated with PF4708671 (20 μM). PF4708671 alone had no significant effect on the amplitude or frequency of IPSCs and EPSCs (Fig. 5, Tables 1 and 2) but slightly reduced the EPSC decay time (by 20%, *t*_24_= 2.46, *P*= 0.021, n=12 neurons from 5 mice, Fig. 5, Table 2). In the presence of PF4708671, Aβ_1-42_ did not affect IPSC amplitude or the frequency of IPSCs or EPSCs (Tables 1 and 2), but slightly reduced the EPSC decay time (by 22%, *t*_23_= 2.24, *P*= 0.035, n=14 neurons from 6 mice, Table 2). These data suggest that the Aβ_1-42_-induced changes to IPSC and EPSC properties are mediated through mTOR/S6K1 activation.

### Degeneration of hippocampal γ-oscillations in APP/PS1 mice and rescue by inhibiting mTOR/S6K1 signaling

Gamma oscillations are markedly reduced in patients diagnosed with AD [59]. To test whether γ-oscillations are affected in an AD animal model that over-expresses Aβ, we compared γ-oscillations induced by 100 nM kainate in slices from APP/PS1 mice (4-6-month-old) with those from age-matched wild-type mice (Fig. 6A, B). The γ power of the oscillations recorded in slices from APP/PS1 mice was 64% lower than that recorded in slices from wildtype mice (Mann-Whitney U test *P*= 0.008, n= 18 slices from 18 mice for WT, n= 15 slices from 15 mice for APP/PS1 mice, Fig. 6A-C) and the peak frequency was higher (by 3%, Mann-Whitney U test *P*< 0.05, Fig. 6D). Whereas the γ-oscillation in slices of wildtype mice was very regular, reflected by peaks of 0.42±0.05 at 32±1 ms in the auto-correlogram, the autocorrelation peak of APP/PS1 mice was smaller (0.29±0.03, *t*_31_= 2.141, *P*= 0.040) at 29±1 ms (example in Fig. 6E), indicating that the oscillation was less regular.

**Figure 7.**
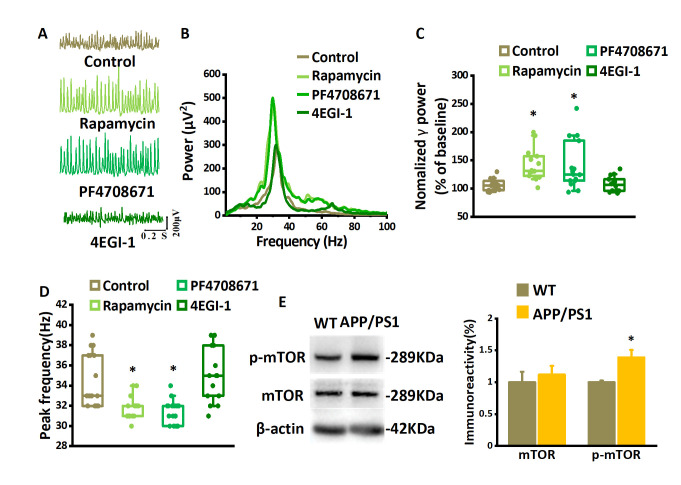
**The suppression of γ-oscillations in APP/PS1 mice depends on mTOR/S6K1 pathway hyper-activation**. Example of traces. (**A**) and power spectra (B) of γ-oscillations recorded in slices from APP/PS1 mouse without and with the application of rapamycin, PF4708671, or 4EGI-1. (**C**) γ-oscillation power in APP/PS1 mice as % of baseline control, rapamycin, PF4708671 or 4EGI-1. n=15 for control, n=14 for rapamycin, n=15 for PF4708671 and n=14 for 4EGI-1. (**D**) γ-oscillation peak frequency in APP/PS1 mice in control and after rapamycin, PF4708671, or 4EGI-1. Details as in (C). Data are presented as median±min-max, *, *P*< 0.05, analyzed by Mann-Whitney U test. (**E**) Representative Western blot quantification of mTOR and the serine 2448 phosphorylated mTOR (p-mTOR). Relative protein level was calculated by normalizing phosphorylated form levels to their corresponding total protein levels and total protein levels to the β-actin levels, respectively. Data are presented as mean ± SEM. *, *P*< 0.05, compared with WT, t-test, n=12 mice for each group.

Because inhibition of the mTOR/S6K1 pathway could prevent the Aβ_1-42_-induced reduction of γ-oscillations, we predicted that inhibition of the mTOR/S6K1 pathway would restore γ-oscillations in APP/PS1 mice. To test this, 200 nM rapamycin or 20 μM PF4708671 were applied after the kainate-induced γ-oscillations were established for 60 minutes (Fig. 7A). Compared with the APP/PS1 control, the application of rapamycin increased γ power (example in Fig. 7A and B) by 25% (the control γ power was 105% of baseline, *P*< 0.05, Mann-Whitney U test, n= 15 slices from 15 mice for control, n= 14 slices from 14 mice for rapamycin, Fig. 7C) and reduced the peak frequency by 3% (*P*< 0.05, Mann-Whitney U test, Fig. 7D). Compared with the APP/PS1 control, the application of PF4708671 increased the γ power (example in Fig. 7A and B) by 19% (*P*< 0.05, Mann-Whitney U test, n=15 slices from 15 mice for PF4708671, Fig. 7C) and reduced the peak frequency by 3% (*P*< 0.05, Mann-Whitney U test, Fig. 7D). To determine the role of eIF4E-eIF4G interactions in the suppression of γ-oscillations in APP/PS1 mice, we also tested the effect of 4EGI-1 on γ-oscillations. 4EGI-1 (50 μM, application for 60 min) had no significant effect on γ power (increased by 1% compared with the control, *P*< 0.05, Mann-Whitney U test, n= 14 slices from 14 mice, Fig. 7A, B and C) or the peak frequency (increased by 6% compared with the control, *P*< 0.05, Mann-Whitney U test, Fig. 7D). This suggests that the degradation of γ oscillations in APP/PS1 mice is partly caused by an over-activity of the mTOR/S6K1 pathway.

To examine whether mTOR was over-activated in APP/PS1 mice, hippocampal CA3 tissue was dissected for Western blotting. Total mTOR levels were not different between APP/PS1 and wild-type mice (t_4_= 0.551, *P*= 0.601, each sample collected from 3 mini slices of hippocampal CA3 region of 3 mice, n= 4 independent experiments from 12 mice, Fig. 7E). However, compared to that in wildtype, mTOR serine 2448 phosphorylation in CA3 of APP/PS1 mice was increased by 38% (t_4_= 3.216, *P*= 0.018, Fig. 7E). The increased mTOR serine 2448 phosphorylation was strikingly similar to the increase induced by Aβ_1-42_ in wildtype slices (Fig. 3E) and suggests that the mTOR/S6K1 pathway mediates the disruption of γ-oscillations in AD.

## DISCUSSION

In this study, we investigated the role of oligomeric Aβ_1-42_ in modulating hippocampal γ-oscillations *in vitro*. Aβ_1-42_ strongly suppressed γ-oscillations while increasing the peak frequency. Aβ_1-42_-induced hyper-activation of mTOR was both necessary and sufficient to suppress γ-oscillations through activation of S6K1 rather than through inhibition of eIF4E. Activation of the mTOR/S6K1 pathway decreased spontaneous EPSC amplitude and frequency and IPSC frequency. Inhibition of the mTOR/S6K1 pathway rescued the impairment of hippocampal γ-oscillations in APP/PS1 mice.

Oligomeric Aβ_1-42_ is increased in the brain tissue of AD patients and animal models [60]. Because increased levels of oligomer Aβ_1-42_ are observed early in the development of AD, it has been the target of AD therapies [61, 62].

The oligomeric Aβ_1-42_ concentrations that were effective in suppressing γ-oscillations are within the concentration range of oligomeric Aβ, found in wet brain tissue of AD patients [53]. Still, no information is available about the oligomeric Aβ_1-42_ concentration in the extracellular space. Aβ_1-42_ was more potent in its oligomeric or fibrillar form than as monomers, which confirms a study by Kurudenkandy et al.[25] and is in line with the increased toxicity of the oligomeric form [63, 64]. Although the fibrillar Aβ_1-42_ was equally potent as the oligomeric form, it is unlikely that large fibrils can affect γ-oscillation-generating networks directly [25] because the average extracellular space is only ~50 nm [51]. Most likely, fibrils are turned into oligomers in the tissue through a fibril-catalyzed secondary nucleation reaction [52]. The < 10 nm diameter oligomers will probably diffuse into the tissue and affect the γ-generating networks.

A similar impairment of γ-oscillations was observed in the 4-month-old APP/PS1 mouse [16] and has been described at very early stages in other AD animal models [20, 21, 23]. Early-stage AD is associated with the hyper-activation of mTOR in patients and animal models [36, 37]. Oligomeric Aβ_1-42_ caused a hyper-activation of mTOR that was necessary for the effect of Aβ_1-42_ on γ-oscillations, as shown by the effect of the mTORC1 inhibitor rapamycin. Hyperactivation of mTOR was also demonstrated in the hippocampus of 4-month-old APP/PS1 mice. Rescuing impaired γ-oscillations in slices from APP/PS1 mice by rapamycin confirms that mTOR1 hyper-activation is necessary for the Aβ_1-42_ effect on γ-oscillations. Hyperactivation of mTORC1 was also sufficient for the Aβ_1-42_-induced impairment of γ-oscillations, since activating the mTOR1 pathway in neurons by leucine [65], could mimic the Aβ_1-42_ effect. The rapamycin-sensitive mTORC1 suppresses autophagy and promotes protein synthesis by phosphorylating S6K1 and 4E-BPs [66]. Whether the acute impact of Aβ_1-42_ on γ-oscillations is caused by suppression of autophagy remains to be determined. Our results identify the mTOR/S6K1 pathway as responsible for the suppression of γ-oscillations. Because eIF4E phosphorylation levels are elevated significantly in the later stages of AD, where they are associated with tau hyper-phosphorylation [56], the mTOR/S6K1-induced suppression of γ-oscillations by oligomeric Aβ_1-42_ may be instrumental in the cognitive deficits, especially in the early stages of AD. In addition, a lack of γ-oscillations was proposed to contribute to the development of AD-associated pathology [23].

The suppression of γ-oscillations by mTOR hyperactivation, whether by Aβ_1-42_, leucine or in the APP/PS1 AD model, was invariably accompanied by an increase in peak frequency, which was reduced by rapamycin that restored γ power as well. This inverse relation between γ power and peak frequency is expected since the IPSC amplitude determines the cycle length [67]. It suggests that the Aβ_1-42_-induced reduction in power and increase in the frequency of γ-oscillations, results from a reduced amplitude of the combined IPSCs of interneurons contributing to the synchronization. However, mTOR hyper-activation did not affect the amplitude of spontaneous (not rhythmic) IPSCs, indicating normal GABAergic synaptic transmission. The mTOR/S6K1-induced reduction of IPSC frequency points to a reduced presynaptic GABA release probability. This can be due to a reduction of AMPA receptor-mediated fast EPSCs on interneurons. Fuchs et al. [10] showed that a reduced AMPA receptor-mediated recruitment of parvalbumin-expressing interneurons caused a reduction of γ power, an increase in peak frequency, and a reduction in regularity, a pattern identical to the Aβ_1-42_ effect on γ-oscillations. The Aβ_1-42_-induced reduced amplitude and frequency of spontaneous EPSCs in CA3 pyramidal neurons can explain a reduced interneuron activation. Our results are in line with the observations of Ramirez et al.[68], who showed that rapamycin increases the frequency of miniature EPSCs of rat hippocampal primary neurons by modulating neurotransmitter release [68]. Interestingly, a decreased glutamate release probability and a reduced spontaneous EPSC frequency were also observed in the CA3 region of 4-month-old APP/PS1 mice [69, 70]. Our observations differ from those of Kurudenkandy et al. [25], who report increased EPSC amplitude and frequency and decreased IPSC amplitude and frequency. However, they tested a high concentration (1 µM) of the fibrillar form Aβ_1-42_, producing an unknown concentration of oligomer Aβ_1-42_ [52]. This advocates using a controlled oligomer preparation [44, 45].

Alternatively, the mTOR hyper-activity-induced suppression of γ-oscillations may be caused by reduced intrinsic excitability of interneurons. Supporting this hypothesis, Verret et al. [22] reported a reduction in γ-oscillations in human APP mice and AD patients, linked to decreased levels of the interneuron-specific voltage-gated sodium channel subunit Nav1.1 in parvalbumin-expressing interneuron. Reduced intrinsic excitability of interneurons can also result from a metabolic impairment of fast-firing interneurons that are very energy-demanding during γ-oscillations. Gamma oscillations are exquisitely sensitive to metabolic stress [71] and more vulnerable in early AD [72]. mTOR hyper-activation affects metabolic processes involved in energy homeostasis [32, 33] and impairs mitochondrial function [35], which reduces interneuron activity [31, 34] and leads to reduced hippocampal γ-oscillations in aged mice [31].

The mTOR hyper-activation-induced impairment of γ-oscillations is likely to contribute to the cognitive impairment observed in early-stage AD and MCI since γ activity serves as an elementary operator of brain function and communication, as reviewed by Basar [73]. Impairment of γ activity has been related to the impairment of working memory [10], spatial reference memory [11], and context-dependent memory deficits [22], cognitive deficits typical for early stages of AD.

Our study does not exclude other ways by which mTOR hyperactivity can contribute to cognitive deficits [74], but restoring γ-oscillations is a potential target for early-stage AD therapy. For instance, restoring the intrinsic excitability of parvalbumin-expressing interneurons, increased γ-oscillations, and reversed cognitive impairment in the human APP AD model [22]. Interestingly, restoring degenerated γ-oscillations in an AD model by optogenetically driving parvalbumin-expressing interneurons reduced Aβ accumulation [23], suggesting that restoration of γ-oscillations may have not only short-term cognitive benefits but can even attenuate AD-associated pathology.
